# Obstacle detection for lake-deployed autonomous surface vehicles using RGB imagery

**DOI:** 10.1371/journal.pone.0205319

**Published:** 2018-10-22

**Authors:** Philippe Paccaud, D. A. Barry

**Affiliations:** Laboratoire de technologie écologique (ECOL), Institut d’ingénierie de l’environnement (IIE), Faculté de l’environnement naturel, architectural et construit (ENAC), Ecole polytechnique fédérale de Lausanne (EPFL), 1015 Lausanne, Switzerland; University of Kentucky, UNITED STATES

## Abstract

We describe and test an obstacle-detection system for small, lake-deployed autonomous surface vehicles (ASVs) that relies on a low-cost, consumer-grade camera and runs on a single-board computer. A key feature of lakes that must be accounted for is the frequent presence of the shoreline in images as well as the land-sky boundary. These particularities, along with variable weather conditions, result in a wide range of scene variations, including the possible presence of glint. The implemented algorithm is based on two main steps. First, possible obstacles are detected using an innovative gradient-based image processing algorithm developed especially for a camera with a low viewing angle to the water (i.e., the situation for a small ASV). Then, true and false positives are differentiated using correlation-based multi-frame analysis. The algorithm was tested extensively on a small ASV deployed in Lake Geneva. Under operational conditions, the algorithm processed 640×480-pixel images from a Raspberry Pi Camera at about 3—4 Hz on a Raspberry Pi 3 Model B computer. The present algorithm demonstrates that single-board computers can be used for effective and low-cost obstacle detection systems for ASVs operating in variable lake conditions.

## Introduction

Autonomous Surface Vehicles (ASVs) are increasingly deployed in different applications, including for acquisition of field data and/or for monitoring inland water bodies, in particular lakes. Compared with oceans, lakes are typically low energy, enclosed environments, with a potentially higher density of floating obstacles (e.g., birds, people or boats). Detection of such obstacles is a basic requirement for ASVs to navigate lakes safely in fully autonomous, i.e., unsupervised, mode. In addition, in supervised mode, where an operator observes the ASV during deployments, automated obstacle detection assists the operator in ensuring the safe operation of the ASV.

While the problem of obstacle detection for lake-deployed ASVs is relatively new, the same problem for Unmanned Ground Vehicles (UGVs) is long-standing. Some of the solutions implemented for ASVs are thus inspired from experience acquired on UGVs. Sensors used first on UGVs and subsequently on ASVs for detecting obstacles are LIDARs (Light Imaging, Detection And Ranging), monocular and stereo cameras, SONARs (SOund Navigation And Ranging) and RADARs (RAdio Detection And Ranging).

Halterman et al. [1] obtained excellent results using a Velodyne HDL-64E LIDAR initially developed for UGVs. Even though scanning LIDARs allow for highly reliable obstacle detection, their cost is relatively high, and the range is limited. Sorbara et al. [2] tried to overcome the range and cost problem of scanning LIDARs by developing an embedded sensor consisting of a single beam long-range Laser Range Finder (LRF), mounted on an actuated platform, along with RGB (red-green-blue) and thermal cameras. The cameras were used to detect possible obstacles, with their distances measured by the LRF.

RADARs, even though their range is excellent, are bulky and thus more suitable for big vessels. They perform poorly in detecting small targets at small distances [3]. Apart from their expense, they are also usually used under human supervision.

Other widely used solutions are based on stereo vision cameras. Cameras have the advantage of being cheap and lightweight, thus they can easily be used even for the small ASVs typically used on lakes. Furthermore, a camera’s power consumption is low compared to most other range sensors. Small ASVs typically run on batteries and so power consumption is a major factor affecting the autonomy duration. Neves et al. [4] implemented stereo vision abilities on an ASV using a single-board computer, but detection was limited to predefined targets and buoys. Wang et al. [5] also used a stereo vision system, but chose a different approach. They used the output of one of the cameras to detect obstacles and then estimated its distance from the ASV using stereo vision algorithms [6].

Here, we present an inexpensive real-time obstacle detection system built upon imagery from a mono-vision RGB camera. Besides the low cost, the detection range is essentially only limited by the image resolution, i.e., detections depend on the object being present in at least several pixels. The algorithm used here, similarly to others also based on a single mono-vision camera, consists of two main steps:

Step 1. Here, (potential) obstacles are identified in a given single frame, with a focus on achieving a good recall score (i.e., Eq 1) although at the cost of a decreased precision score (i.e., Eq 2):
  $$recall=\frac{True\;Positive}{True\;Positive\;+\;False\;Negative}$$ (1)
  $$precision=\frac{True\;Positive}{True\;Positive\;+\;False\;Positive}$$ (2)
Step 2. To discriminate between true positives and (temporary) false positives, a multi-frame analysis is performed. This step assumes that true obstacles will vary slowly between two consecutive frames, keeping some characteristics (e.g., pixel size, position) almost constant, allowing tracking of those objects through multiple frames.

Different implementations of this strategy are possible, some of which are reviewed here. Oren Gal [7] used an occurrence matrix to segment the possible obstacles from the water pattern and tried to find the correspondence between feature points using two consecutive frames. Guo et al. [8], whose ASV was a sailboat, computed the difference between two consecutive frames assuming that obstacles moved relatively to the sailboat. Then, a cluster in the RGB-space was created from the remaining constant pixels. Changed pixels, not fitting the computed cluster, were assumed to correspond to obstacles. No information was given about the time difference used to compute the variation image, nor how the cluster was computed.

For their monocular analysis, Wang et al. [5] implemented a saliency filter to detect possible obstacles. Then, a Harris corner detector was used to find feature points that were fed to a pyramidal implementation of the Lucas-Kanade feature tracker. The objects whose feature points could be consistently tracked for a few consecutive frames were assumed to be obstacles. In a different approach, Kristan et al. [9] presented a single-step algorithm wherein they fitted a Gaussian Mixture Model (GMM) to a given frame to segment water, land and sky regions. The GMM relies on the availability of priors that are pre-computed using training data. Given the range of conditions and environments hosting lakes, site-specific training data would be required for good algorithm performance, and the likelihood of poor performance would increase under untrained conditions.

In a non-ASV application, Fefilatyev and Goldgof [10] presented an algorithm that monitored ship traffic in open sea from a stationary buoy. The low height of the camera above the water surface allowed them to assume that objects of interest crossed the sea-sky horizon (no land existed in the considered scenes). The multi-frame analysis then sorted between false positives due to high waves and true positives.

The aforementioned obstacle-detection methods and algorithms were considered for use on an existing small ASV used for physical limnology research on Lake Geneva (local name lac Léman), located on the border of France and Switzerland. The energy-efficient system presented here uses low-cost off-the-shelf components. It is configured as a standalone module that can be easily interfaced with existing platforms. The ASV used in this work is described elsewhere [11]. It provides power as well as continuous communication and monitoring over the 4G cellular network using the TCP/IP (i.e., internet, Transmission Control Protocol/Internet Protocol) protocol. The communication system permits, if desired, video steaming over the internet in addition to obstacle detection. To satisfy the cost and energy consumption requirements, we selected a Raspberry Pi computer and a single camera module (i.e., a monocular vision hardware solution).

We tested, when appropriate, the different monocular vision algorithms described above. Oren Gal’s [7] image segmentation gave poor results, most probably due to the difference of scenes considered for the algorithm. In our case, waves are much more contrasted, with highly non-periodic patterns. Based on the presented frames, we concluded that the most probable cause for the poor performance was that they placed their camera much higher than our system could allow, limiting the use of the algorithm to medium/large sized ASVs.

Guo et al. [8] provided little information on their algorithm, and it was demonstrated only on bright yellow buoys, not arbitrary obstacles.

The solution of Fefilatyev and Goldgof [10] cannot be implemented in a lake. Because their application is in the open sea, they assume that everything above the horizon could be an obstacle, whereas in a lake it is very common to see the shore above the horizon.

The prior training needed for the algorithm of Kristan et al. [9] is a major drawback. Not only did we wish to avoid a (costly) training step, the amount of training is significant when there is high scene variability, and when different lighting conditions must be considered.

The solution of Wang et al. [5] was found to be promising. In testing, we found that their horizon-detection algorithm regularly failed—this step is necessary to restrict where obstacles are possibly located in an image. One reason is that they assume that the horizon can be anywhere within an image. High gradients, with which they locate the horizon in the frame, can also be found at the shore-sky limit or in the horizon’s reflections on the water surface. Such false positives are common if the water surface is flat with few high-frequency perturbations. Also, their algorithm ran slowly on our low-power computer due to their use of a saliency filter [12], a Harris corner extraction, and a pyramidal Lucas-Kanade feature tracker on every frame. The saliency filter step was particularly slow due to the L*a*b color-space transformation. Furthermore, compared with our solution, their solution is expensive, as it used two Point Grey Grasshopper CCD cameras, each costing over US $1000. By comparison, the cost of the presented system is at least an order of magnitude less.

In the following, we present an image-based obstacle detection algorithm for small ASVs deployed on lakes. The algorithm’s performance is demonstrated using, as mentioned, a low-cost computer and camera that give a system capable of fast obstacle detection in varied conditions and without prior training.

## Algorithm

Algorithm 1 presents the pseudo code of the whole procedure. The main steps of the procedure consist of locating the water-shore or water-sky horizon in the frame, segmenting the image below the horizon to extract potential obstacles, and comparing the result with previous frames to filter false positives.

**Algorithm 1**. **Obstacle detection main loop**. Pseudo code of the obstacle detection procedure.

1: **procedure** ObstacleDetection

2:  *listOfBlobs* ← *initializeEmpty*()

3:  **while** True **do**

4:   *frame* ← *getNewFrame*()

5:   *attitude* ← *getAsvAttitude*()

6:   *horizon* ← *findHorizon*(*frame*, *attitude*)

7:   *croppedFrame* ← *cropAboveHorizon*(*frame*, *horizon*)

8:   *segmentedFrame* ← *imageSegmentation*(*croppedFrame*)

9:   *listOfBlobs*.*newBlobs* ← *getBlobsFromFrame*(*segmentedFrame*)

10:   *listOfBlobs* ← *trackObstacles*(*listOfBlobs*)

11:   *listOfBlobs*.*previousBlobs* ← *listOfBlobs*.*newBlobs*

12:   **if** *listOfBlobs*.*obstacleBlobs* ≠ *emptyList* **then**

13:    *sendObstacleListToNavigationSystem*(*listOfBlobs*.*obstacleBlobs*)

### Region of interest

The first step is locating the appropriate region of interest (ROI) in the image. Limiting the detection to a ROI not only reduces the computation time, but also limits false positives that arise in examining heterogeneous parts on an image. For obstacle detection from ASVs, we assume that obstacles will be located on the water surface. Thus, as in most other algorithms, we first detect the horizon, or more specifically the water-land or water-sky demarcation. Once detected, the horizon separates the image into two regions, water and above. The water region requires a different treatment than the rest of the image (e.g., different parameters [5]) and, indeed, as is the case here, might be the only region analyzed [7], which results in more efficient analysis.

In most cases, horizon detection algorithms rely on either a statistical method or on an edge analysis. For the former, the horizon is defined as the line that best separates the sky and water and minimizes their relative intra-class variance [10]. More sophisticated methods can also take the shore into consideration but rely on learning algorithms [9], which we avoid here.

**Fig 1:**
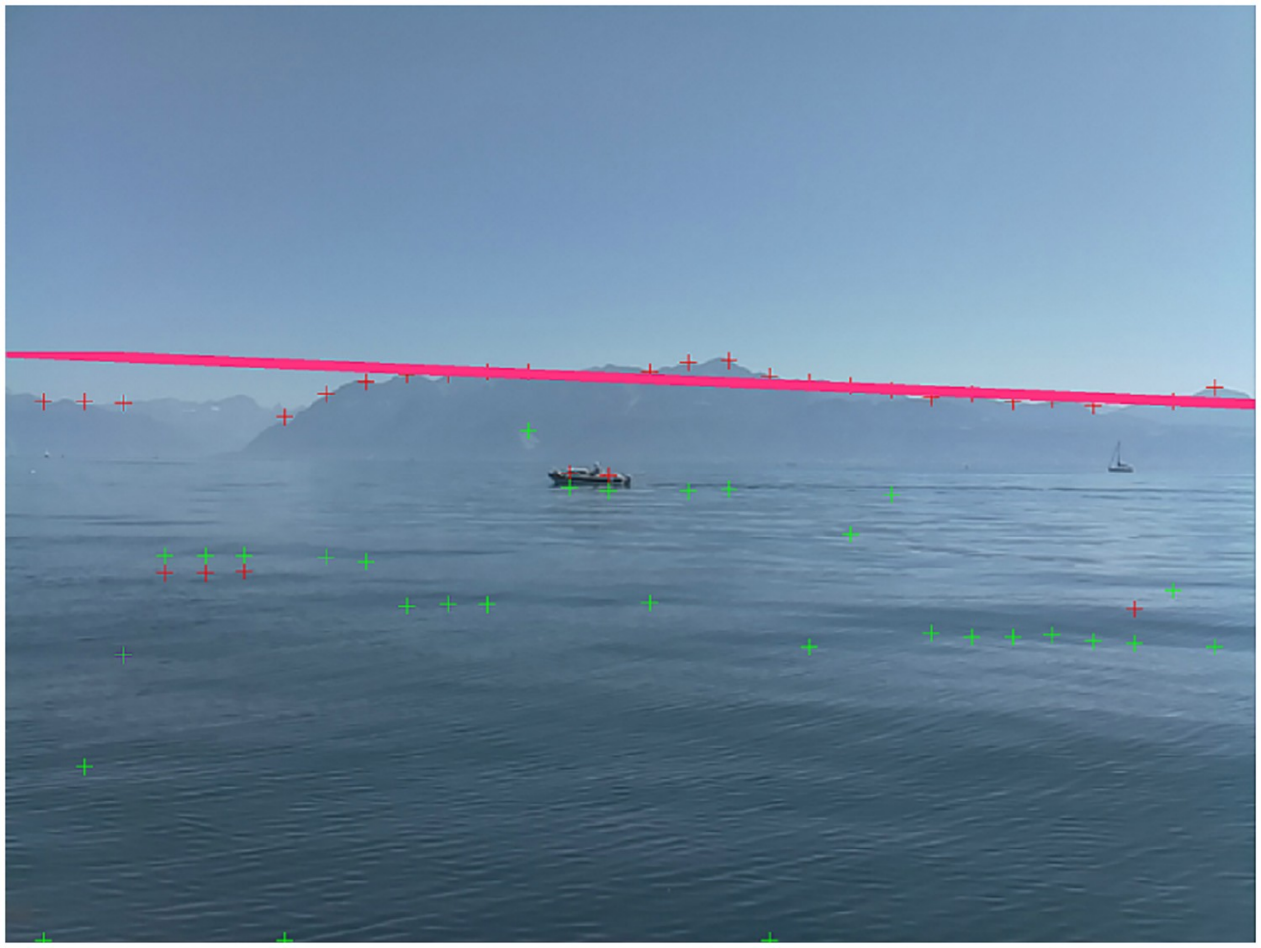
Failed horizon detection due to haze. Example of failed horizon detection using the full-frame RANSAC method (see text). The image quality at the water-land boundary is compromised due to haze. Consequently, none of the maximum positive and negative gradient points (red and green crosses, respectively) are located on the actual horizon.

For the latter, the horizon is assumed to be the most salient line in the frame. For instance, to detect it, a Canny edge detection followed by a Hough transform can be used [7]. Another algorithm [5] finds the most positive and most negative gradient positions along multiple vertical stripes in the image and fits a line through these points using the RANSAC (RANdom SAmple Consensus) algorithm [13]. This method will be refered to as the full-frame RANSAC method below. However, all these solutions can fail in cases where the horizon is not well defined (e.g., due to haze, poor lighting or adverse weather conditions), such as the case shown in Fig 1. Also, in the particular case of lakes, land is often seen above the water, meaning that the land-sky demarcation is significant. This demarcation can, if it is relatively straight, lead to a false horizon detection due to the strong gradient across it.

Fig 1 shows a situation where image analysis alone cannot be expected to detect the land-water boundary reliably. However, with the widespread availability of (inexpensive) Inertial Navigation Systems (INSs), it is feasible to track the camera’s attitude in real time, and use this information to improve horizon detection. Of course, any ASV will incorporate an INS, so the required information is available. Based on the ASV’s INS data, and the placement of the camera on the ASV, we compute the horizon line corresponding to its defined distance, *d*, for measured pitch (*θ*) and roll (*φ*) angles. The vertical pixel position, *p*_*y*_, of the horizon for a given horizontal pixel position, *p*_*x*_, can be approximated using:

$$p_{y}=round[\frac{Res_{v}}{FOV_{v}}({\text{tan}}^{-1}(\frac{h}{d})+\theta )-\text{tan}\left(\varphi \right)(p_{x}-\frac{Res_{h}}{2})+\frac{Res_{v}}{2}],$$ (3)

where *Res*_*h*_ and *Res*_*v*_ are, respectively, the vertical and horizontal resolutions of the camera, *FOV*_*v*_ the vertical field of view, and *h* is the height of the lens above the water plane. The pixel position (*p*_*x*_, *p*_*y*_)^*T*^ uses the standard image coordinate system with top-left origin, horizontal *x*-axis, and vertical *y*-axis. The positive direction of the intrinsic ZYX Tait-Bryan angles *θ* and *φ* is derived from the right-hand rule using the standard aircraft reference frame applied to the ASV. Increasing the distance of the horizon *d*, reduces its influence on the vertical pixel position *p*_*y*_. In our application, to relax the dependency on the unkown horizon distance *d*, we assume that *d* ≫ *h* and so ${\text{tan}}^{-1}(\frac{h}{d})\approx 0$.

The attitude-based horizon detection can be used in any condition but, due to small errors in attitude estimation, is less precise than for images where the water-land boundary is clear. So, using the attitude estimation and its possible angular error, we define an ROI in the image in which the horizon is located. To determine the vertical size of the ROI around the estimated horizon, we assumed a maximum attitude error of 1° along each axis (this value can be adapted to the precision of the INS data). The resulting window size *w* is computed using:

$$\frac{w}{2}=round(\frac{FOV_{v}}{Res_{v}}\theta_{error}+\text{tan}(\left|\phi \right|+\phi_{error})\frac{Res_{h}}{2})$$ (4)

where *θ*_*error*_ = *ϕ*_*error*_ = 1°, converted to radians. We then apply the RANSAC method twice, to potentially detect both water-land and land-sky horizons, as they could both be present in an image. Three different cases are possible:

- If no horizon is detected with the RANSAC algorithm, we use the horizon estimated with the ASV’s attitude. Since no detection probably means that the horizon is blurred (e.g., by haze), any possible land that might be included in the analyzed ROI will be blurred too, in which case erroneous detection of obstacles is unlikely (resulting in low risk of false positives). It is also possible that the water-land horizon is actually below the considered attitude-based horizon, meaning that the ASV is close to the shore, in which case the shore is a potential obstacle and must be detected.
- If one horizon is detected, we apply a test (below) to compare it with the horizon determined from the attitude and thereby ascertain if it is a false positive or the actual horizon.
- If two horizons are found, the just-mentioned test is applied to both horizons and most consistent result, if any, is used.

This test uses the slope and the vertical position of the horizon to assess its validity. If the test results in inconsistency of detected horizon(s), the attitude-based horizon is used.

The advantage of this combined (image- and attitude-based) method is that it can still be used when regular horizon detection methods fail, although at the cost of a slightly degraded precision. It is also as precise as gradient-based horizon detection when the horizon can be detected in images.

### Image segmentation

With the horizon detected, the ROI within which obstacles are to be detected is the part of the image lying below the horizon. The next step is to apply an image segmentation algorithm in this ROI to detect possible obstacles. For clarity, we use the following standard image-processing convention. The origin of the reference frame of an image is located at its top-left pixel, with the *x*-axis being horizontal, and the *y*-axis being vertical. Thus, the *x*- or *y*-axis gradient is computed along the specified axis, with the gradient estimated using the Sobel operator.

**Fig 2:**
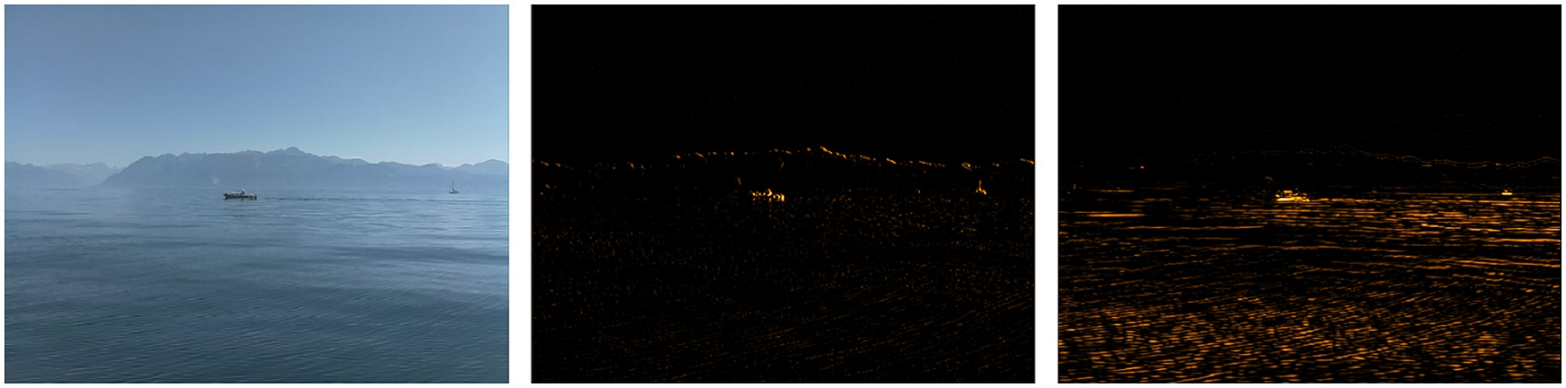
Gradient along *x*- and *y*-axis comparison. Normalized Sobel gradient for a given frame (left) along the *x*-axis (middle) and *y*-axis (right). The two boats (i.e., obstacles) are more readily detected using the gradient along the *x*-axis. Gradients are displayed using a color map for (visual) emphasis.

The presented segmentation algorithm recognizes that the gradient within the ROI is due either to water surface variability (waves or reflections) or to an obstacle, the difference being that the obstacle will be (partially) above the water surface plane. The algorithm uses the fact that the water surface patterns will be squeezed along the optical axis of the camera when projected onto the camera sensor, an effect which is more marked with increasing distance, or equivalently with reducing viewing angle. Hence, scaling of water patterns in the image will be different along vertical and horizontal axes. Obstacles rising above the water surface will only undergo symmetrical scaling. Consequently, the *x*-axis gradient can be used as a metric to distinguish surface water patterns from obstacles. This is apparent in Fig 2 where the *x*-axis gradient is influenced by the presence of obstacles whereas the *y*-axis gradient is mainly controlled by water patterns.

Sun glint is common in low-angle images, and obstacles within images can be difficult to detect. Glint-filled pixels are saturated, causing high gradients with surrounding pixels. By reducing the intensity of these pixels, the gradients will also be reduced, and consequently obstacles within the glint will be more easily detected.

The presence of glint in a given frame is determined using the following metric. Each image is segmented using a fixed-value threshold, and the number of separate regions is counted. The basis of this approach is that glint creates a speckled water surface, which is readily detected via thresholding. If more than 20 distinct regions are found, we assume that they are due to glint. For such images, the pixel saturations are limited to a given maximum according to:

$$\begin{array}{c} pixel=\{\begin{array}{ccc} {\left(\frac{r}{max(r,g,b)}t,\frac{g}{max(r,g,b)}t,\frac{b}{max(r,g,b)}t\right)}^{T} & \text{if} & max(r,g,b)>\tau \\ {\left(r,g,b\right)}^{T} & \text{otherwise} \end{array} \end{array}$$ (5)

where *pixel* is the vectorized RGB color-space pixel value (*r*, *g*, *b*)^*T*^, *r*, *g*, and *b* are the red, green, and blue channel values, and *τ* the normalized threshold. It can be shown that this formula is equivalent to a regular threshold on the value channel in the HSV (Hue, Saturation, Value) color space. This step reduces the effect of pixel saturation and leads to improved performance of the subsequent gradient-based detection steps.

**Fig 3:**
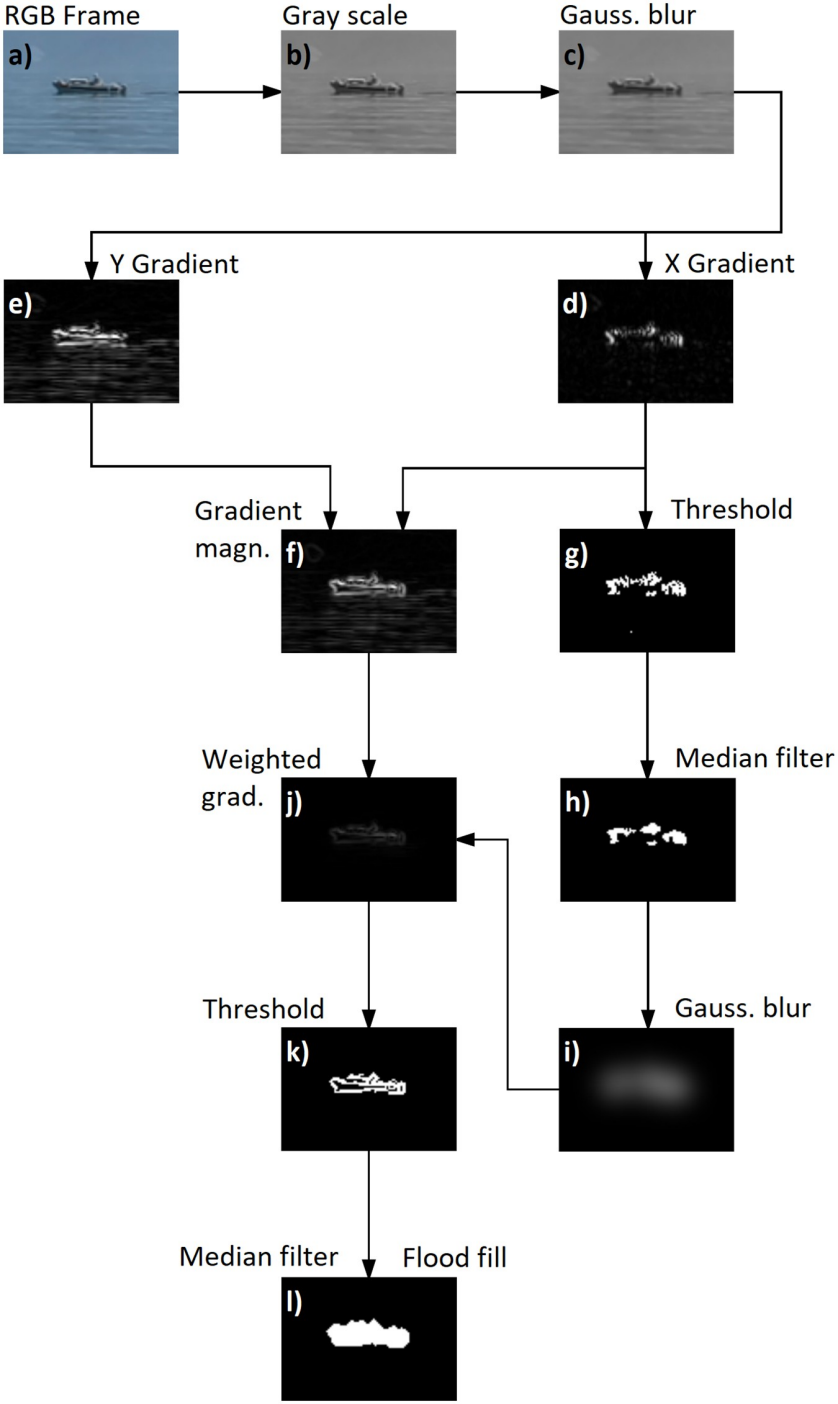
Summary of the image segmentation algorithm. The label corresponds either to the associated frame or to the processing required to obtain that frame. Details of the different steps are provided in the text.

As seen in Fig 3 and Algorithm 2 showing the different steps for the image segmentation and the corresponding pseudo code, we convert the new RGB frame (a) to gray levels (b) and apply a 3 pixel-by-3 pixel Gaussian filter (c). Then, a Sobel operator is used separately along the *x*- and *y*-axes (d-e) to compute the directional gradients. A percentile threshold is applied on the *x*-axis gradient (g). The value of the threshold is chosen so that 0.2% of the pixels are kept in the frame. A minimum value for the threshold is also defined to avoid segmenting noisy, low gradient pixels in images. An advantage of our approach is that the threshold value is not fixed, and so obstacle edges can be detected independently of the scene lighting conditions. A disadvantage is that some edges will be found even if there is no obstacle and also some edges could be missed if there are many obstacles. The minimum threshold value is set at 0.2 (for a non-normalized gradient). Any remaining false positives are identified during the multi-frame analysis step below.

**Algorithm 2**. **Image segmentation algorithm**. Pseudo code of the presented image segmentation algorithm.

1: **function** ImageSegmentation(*croppedFrame*)

2:  *binaryFrame* ← *fixedValueThreshold*(*croppedFrame*)

3:  **if** *connectedComponentLabelling*(*binaryFrame*) > *criterion* **then**

4:   *croppedFrame* ← *hsvThreshold*(*croppedFrame*)

5:  *greyFrame* ← *rgbToGrey*(*croppedFrame*)

6:  *smoothedFrame* ← *gaussianFilter*(*greyFrame*)

7:  *xGradient*, *yGradient*, *magnitudeGradient* ← *sobelGradient*(*smoothedFrame*)

8:  *segmentedFrame* ← *percentileThreshold*(*xGradient*)

9:  *obstacleProbMap* ← *gaussianFilter*(*segmentedFrame*)

10:  *weightedGradient* ← *pixelWiseProduct*(*magnitudeGradient*, *obstacleProbMap*)

11:  *obstacleMap* ← *percentileThreshold*(*weightedGradient*)

12:  **return** *medianFilter*(*obstacleMap*)

Observe that in Fig 3g, the detected blob (i.e., supposed obstacle) after thresholding is inconsistent with the obstacle shape. The shape is refined as follows. Since the pixels passing the threshold locate a potential obstacle, they are used to compute a map of weights for the whole frame. For this, we first apply a median filter (h) and convolve the result with a Gaussian smoothing operator (i). For the latter, a window size of 45×45 pixels and a standard deviation of 8 pixels gives good results for the frame resolution and image quality from our camera. This map is then multiplied element-wise with the gradient magnitude frame (f), resulting in Fig 3j, which enhances the gradient surrounding the previously found pixels (g), and allows for detection of horizontal edges. Finally, another threshold using a percentile of 1.5% segmented pixels (and a minimum threshold value of 0.05) is applied (k), after which a median filter is used to smooth the result, and a 4-neighbors flood filling algorithm is applied (l) to recover blobs for the following step.

### Multi-frame analysis

After possible obstacles are segmented in a frame, blobs are processed to filter false positives. False positives mainly arise from irregular water surface reflections. Blobs (i.e., potential obstacles) arising from these causes are distinguished from the actual obstacles using temporal analysis. As mentioned by Wang et al. [5], obstacles undergo regular and consistent motion, whereas reflections are irregular and hence intermittent, and so persist only for short sequences of frames at most. Such false positives can therefore be identified by tracking the blobs within consecutive frames.

To track the blobs, the algorithm first records their binary image, pixel count, and centroid position in each frame. Comparing blobs across frames permits their sorting into four categories:

- New Blobs (NBs)—blobs in the current frame
- Previous Blobs (PBs)—unassigned blobs found in the previous frame(s)
- Possible Obstacle Blobs (POBs)—possible obstacles in the current frame
- Obstacle Blobs (OBs)—blobs in the current frame that are already designated as obstacles

POBs and OBs are recorded for more than two consecutive frames to account for possible tracking failures or poor image segmentation. For each new frame, we compare NBs with OBs, POBs and PBs. If the currently considered blob is a match with a PB, it is recorded as a POB and is given a likelihood parameter. If it matches a POB, the POB’s likelihood value is increased. Above a defined likelihood threshold, a POB becomes an OB. Matched blobs (within one of the categories PB, POB or OB) are assigned the parameters of the NB, which is discarded from the NB list. Any unassigned NBs are labelled as PBs, and the procedure is repeated for the next frame. After each frame, every POB and OB have their likelihood slightly decreased using a defined fixed cost parameter. They are discarded if their likelihood reaches zero.

The correspondence between two blobs is quantified using a confidence value, *c*, which is based on three parameters, given by:

$$\gamma \left(u,v\right)=\frac{\sum_{x,y}[f\left(x,y\right)-{\bar{f}}_{u,v}][t\left(x-u,y-v\right)-\bar{t}]}{\sqrt{\sum_{x,y}{\left[f\left(x,y\right)-{\bar{f}}_{u,v}\right]}^{2}\sum_{x,y}{\left[t\left(x-u,y-v\right)-\bar{t}\right]}^{2}}}$$ (6)

$$s=\frac{min(s_{prev},s_{new})}{max(s_{prev},s_{new})}$$ (7)

$$d=1-{\left(\frac{\sqrt{{\left(c_{prev,x}-c_{new,x}\right)}^{2}+{\left(c_{prev,y}-c_{new,y}\right)}^{2}}}{dist_{limit}}\right)}^{2}$$ (8)

$$c=\frac{max(\gamma )+s+d}{3}$$ (9)

The first parameter, *max*(*γ*), in Eq 9 is the maximum value obtained after 2D normalized cross-correlation of the two blobs (Eq 6). The chosen correlation coefficient is taken from [14], where *f* and *t* represent the reference image and the template, respectively, and $\bar{f}$ and $\bar{t}$ their respective averages for the considered window. The second parameter, *s*, accounts for the difference in the number of pixels within the blobs (Eq 7), with *s*_*new*_ and *s*_*prev*_ being the pixel count of the NB and POB, PB, or OB. The last parameter, *d*, considers the distance between the blobs’ centroid (Eq 8), where *c*_*new*_ and *c*_*prev*_ are the coordinates of the centroid of the blobs and *dist*_*limit*_ is the maximum distance between two blobs for them to be accepted as resulting from the same object. These three parameters are then averaged (Eq 9) to get the confidence value that is compared to a threshold to assess whether the two blobs correspond to the same object.

In practice, the procedure computes the confidence value for each NB lying within the distance *dist*_*limit*_, for every recorded OB. If a match is found, the NB is discarded and the likelihood, *L*, of the OB is increased according to the confidence value and a tunable parameter *η*:

$$L_{new}=L_{old}+\eta c$$ (10)

Based on extensive testing, we found *η* = 3 gave excellent results.

The procedure is then repeated for the POBs and PBs. For the PBs, if a match is found, the blob is assigned as a POB. After this, the likelihood of the OBs and POBs is slightly decreased, and their value is checked to see if some POBs or OBs have been lost (*L* ≤ 0) or if some POBs have become OBs (*L* ≥ 1). Finally, PBs are discarded and the unassigned NBs are set as the PBs for the next frame. The pseudo code is given in Algorithm 3.

**Algorithm 3**. **Multi-frame algorithm**. Pseudo code of the presented multi-frame algorithm.

1: **function** trackObstacles(blobs)

2:  **for all** *newBlob* **in** *blobs*.*nb* **do**

3:   **for all** *obstacleBlob* **in** *blobs*.*ob* **do**

4:    *confidence* ← *blobsMatch*(*newBlob*, *obstacleBlob*)

5:    **if** *confidence* > *confidenceThresholdOb* **then**

6:     *obstacleBlob*.*likelihood* ← *add*(*confidence* × *ratioOb*)

7:   **for all** *possibleObstacleBlob* **in** *blobs*.*pob* **do**

8:    *confidence* ← *blobsMatch*(*newBlob*, *possibleObstacleBlob*)

9:    **if** *confidence* > *confidenceThresholdPob* **then**

10:     *possibleObstacleBlob*.*likelihood* ← *add*(*confidence* × *ratioPob*)

11:   **for all** *previousBlob* **in** *blobs*.*pb* **do**

12:    *confidence* ← *blobsMatch*(*newBlob*, *previousBlob*)

13:    **if** *confidence* > *confidenceThresholdPb* **then**

14:     *blobs*.*pob* ← *addBlob*(*newBlob*, *confidence*)

15:  **for all** *obstacleBlob* **in** *blobs*.*ob* **do**

16:   *obstacleBlob*.*likelihood* ← *subtract*(*likelihoodCostOb*)

17:   **if** *obstacleBlob*.*likelihood* ≤ 0 **then**

18:    *blobs*.*ob* ← *removeBlob*(*obstacleBlob*)

19:   **else if** *obstacleBlob*.*likelihood* ≥ 1 **then**

20:    *obstacleBlob*.*likelihood* ← 1

21:  **for all** *possibleObstacleBlob* **in** *blobs*.*pob* **do**

22:   *possibleObstacleBlob*.*likelihood* ← *subtract*(*likelihoodCostPob*)

23:   **if** *possibleObstacleBlob*.*likelihood* ≤ 0 **then**

24:    *blobs*.*pob* ← *removeBlob*(*possibleObstacleBlob*)

25:   **else if** *possibleObstacleBlob*.*likelihood* ≥ 1 **then**

26:    *blobs*.*ob* ← *addBlob*(*possibleObstacleBlob*, *initialConfidence*)

All the parameters for the matching of NBs with the other labels can be different and tuned separately. This allows obstacles to be tracked more easily once they have been found, without having an increased risk of accepting false positives.

After this procedure, relevant data for detected obstacles (angular position relative to the ASV heading, estimated distance based on the ASV attitude, etc.) are available for ASV navigation.

### Experimental setup

The system was developed then tested in two main steps. In the first step, algorithm tuning and performance assessment were based on numerous video recordings collected from a small motor boat and the forementionned ASV. These recordings were analyzed using the presented algorithm implemented in Matlab. Subsequently, a standalone version was written for field use.

The system hardware consists of a Raspberry Pi embedded computer, a Raspberry Pi Camera, and an Adafruit board interfacing the Bosch BNO055 sensor fusion system. The Raspberry Pi 3 Model B is a single-board computer with a 1.2-GHz 64-bit quad-core ARMv8 CPU and embedded wireless connectivity. The on-board program was written in C++, and makes use of the OpenCV library, a well-known open-source computer vision library. A 5-Mpixel Raspberry Pi Camera Module v1.3 was used to capture the frames. The camera’s full Field Of View (FOV) was measured as 52.3° × 40.8°. Image processing time was improved using a downsampled VGA image resolution of 640 × 480 pixels (image size of about 307k pixels), resulting in a frame rate of about 3—4 Hz. The BNO055 chip computes the attitude of the system at 100 Hz on its internal microcontroller and communicates the resulting Euler angles to the Raspberry Pi. A small camera/BNO055 mounting misalignment was corrected during an initial calibration step.

In the second step, experimental validation was conducted using an autonomous catamaran developed in our laboratory [11]. The platform is about 5-m long by 2.5-m wide, and is embedded with numerous sensors for limnological measurements. This ASV can be either remotely controlled or set to follow a waypoint-defined trajectory in fully autonomous mode. Collection of testing data and validation missions were conducted during summer and autumn of 2016 on Lake Geneva near Lausanne, Switzerland.

**Fig 4:**
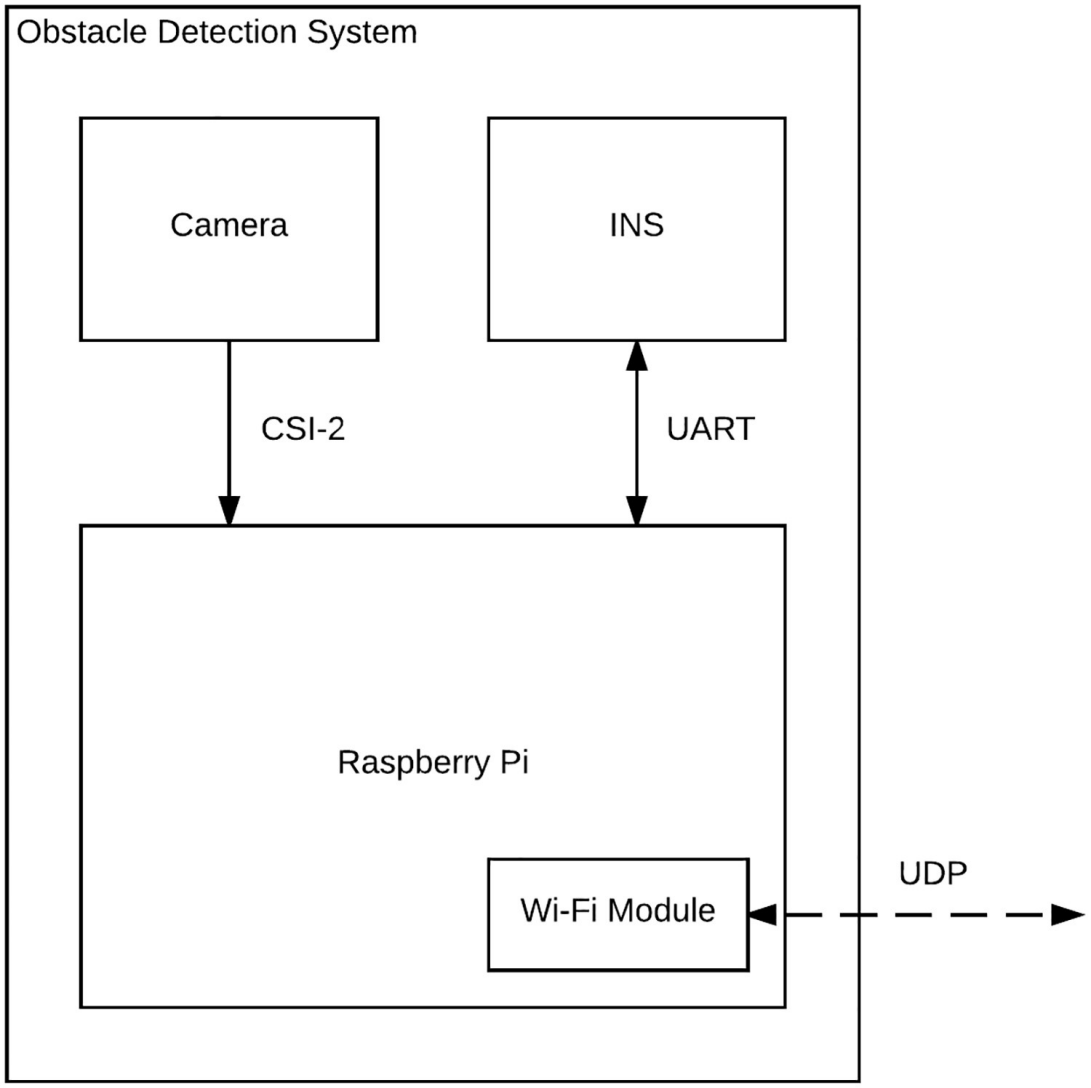
System architecture—Standalone system. The Raspberry Pi computer receives images and attitude information from the Pi camera and INS using CSI 2 (Camera Serial Interface) and UART (Universal Asynchronous Receiver/Transmitter) connections, respectively. After processing the images, obstacle detections are sent to an ASV control module using the User Datagram Protocol (UDP). In a fully integrated system, the images are sent to the ASV’s on-board computer, which receives INS data in any case.

Note that the system can operate in standalone mode (e.g., sending detection alerts to a remote operator), or as integrated into an ASV control system. For the latter, the ASV on-board computer and INS would be used, while a camera is mounted for detecting obstacles. A portable, standalone system was used in our field testing. A wireless network was created using the ASV on-board computer, to which the Raspberry Pi can connect. Once connected, bi-directional communication between the navigation and obstacle detection systems is readily achieved. Fig 4 gives an overview of the hardware architecture of the system as field-tested.

This inexpensive, standalone system has a low power demand. The on-bench power consumption was about 2.7 W (550 mA at 4.97 V). Different scenes necessitate different computational loads. For instance, the attitude of the ASV and/or the number of blobs influence the amount of image processing and multi-frame analysis needed. Image processing computations draw more power as they are partially multi-threaded. Taking the above measured value as typical, the system can run for 3.5 h on a standard single cell 3.7 V, 2600 mAh, 18650 Li-ion battery (without considering voltage conversion loss). Similarly, a 3-cell, 5000 mAh Li-Po battery would power the system for 20.5 h.

Table 1 shows the price of the components (in 2017). The cost of the 3D printed components are omitted, as different ASVs will have different mounting requirements. For the same reason, screws, nuts and jumper cables, which are in any case inexpensive, are not considered.

**Table 1:** Pricing (US$) of the components used for the obstacle detection system. The prices are taken from the Adafruit online store (https://www.adafruit.com/), where all the components can be found.

Component	Price
Raspberry Pi 3—Model B	$39.95
Raspberry Pi Camera Board v2	$29.95
Adafruit IMU Fusion Breakout Board (BNO055)	$34.95
SD/MicroSD Card 8 GB	$9.95
5V/3A Step-down converter, and 2 18650 Li-ion batteries	$29.85
Total	$144.65

**Fig 5:**
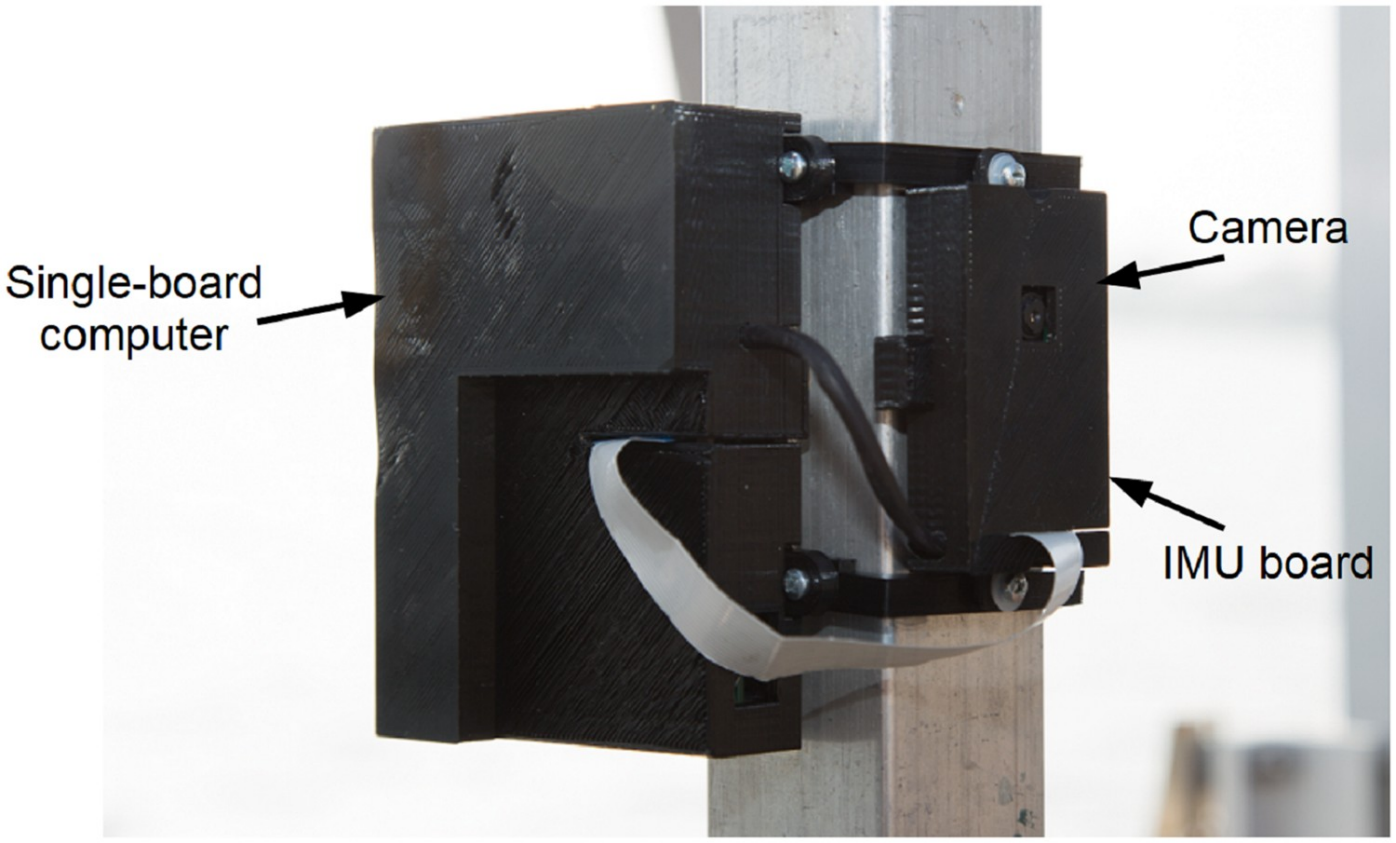
As-tested obstacle detection hardware mounted to the ASV mast.

The assembled device created for our platform is shown in Fig 5. The casing is designed to protect the boards from water splash, which is necessary as the device is placed at about 1 m above water level.

## Results

As mentioned, the algorithm was tested for a range of different scene conditions to assess its efficiency. The results of the different steps of the algorithm are analyzed separately when possible.

### Datasets

**Fig 6:**
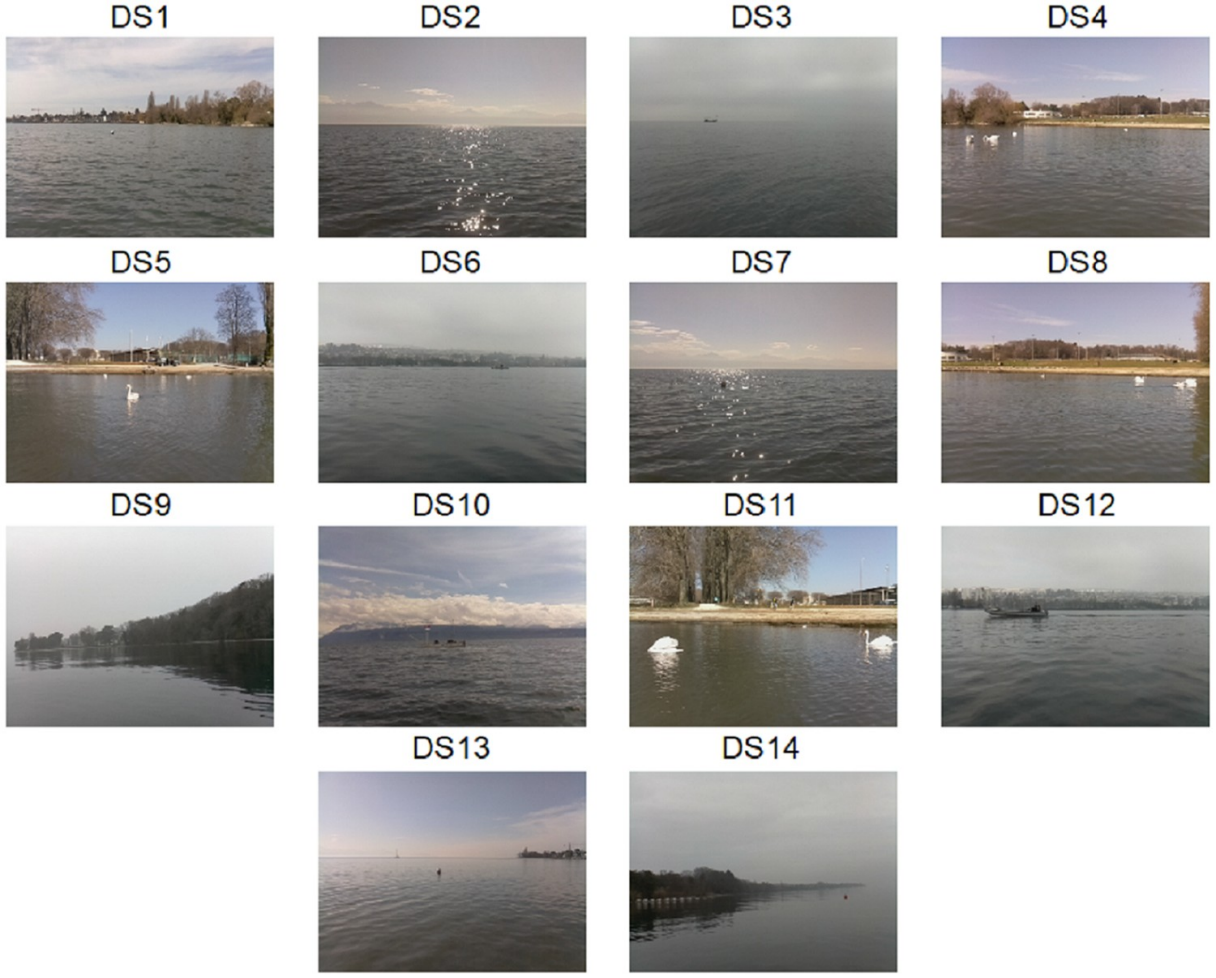
Datasets used to assess the performance of the proposed algorithm. These datasets were recorded with the presented system mounted either on an ASV, or on a private boat.

We used 14 different datasets (DS1—DS14, from video recordings, described in the Table 2) to assess the performance of the proposed algorithm. These datasets, displayed in Fig 6, are available at the Open Science Framework webportal link in the data availability statement. They were selected to represent a wide range of conditions and types of challenges that must be addressed in any obstacle detection algorithm operating on a lake. Note that most of the datasets were obtained using an ASV, as well as a private boat. Authorization to use the ASV on Lake Geneva was given by the *Service des automobiles et de la navigation* of the Canton of Vaud, Switzerland.

**Table 2:** Datasets description. Short description and content of the considered datasets used to compute the results of the proposed algorithm.

Dataset	Nbr of frames	Duration [s]	Description
DS1	327	123	Calm water with a white buoy towards the horizon
DS2	313	113	Calm water with glint, no obstacle with a distant horizon
DS3	230	76	Calm water, cloudy sky, faint horizon and a distant boat
DS4	158	55	Calm water with multiple swans, close to the shore
DS5	122	30	Calm water with multiple swans, close to the shore
DS6	276	78	Calm water, cloudy sky, mid-range distance to the shore, and boat obscured by the shoreline
DS7	307	113	Calm water with glint obscuring a buoy and at far distance from the horizon
DS8	144	50	Calm water with multiple swans, at close range to shore
DS9	201	62	Glassy water, strong shore reflection but no obstacle, mid-range from the shore
DS10	370	118	Rough water partially obscuring a boat, distant from the horizon
DS11	59	15	Calm water with multiple swans, at close range to the shore
DS12	259	91	Calm water, cloudy sky, mid distance from the horizon and a partially-obscured boat
DS13	188	115	Calm water with a white buoy at mid distance from the horizon
DS14	388	115	Glassy water with poor lighting, strong shore reflection and a buoy at mid-range to shore
Total	3342	1154	

### Horizon detection

The cropping of an image, based on attitude estimation, improves the detection of the horizon compared to the standard visual-only full-frame RANSAC method. By reducing the inspection ROI around the estimated position of the horizon, water patterns and the land-sky limit that confound horizon detection are not detected.

**Fig 7:**
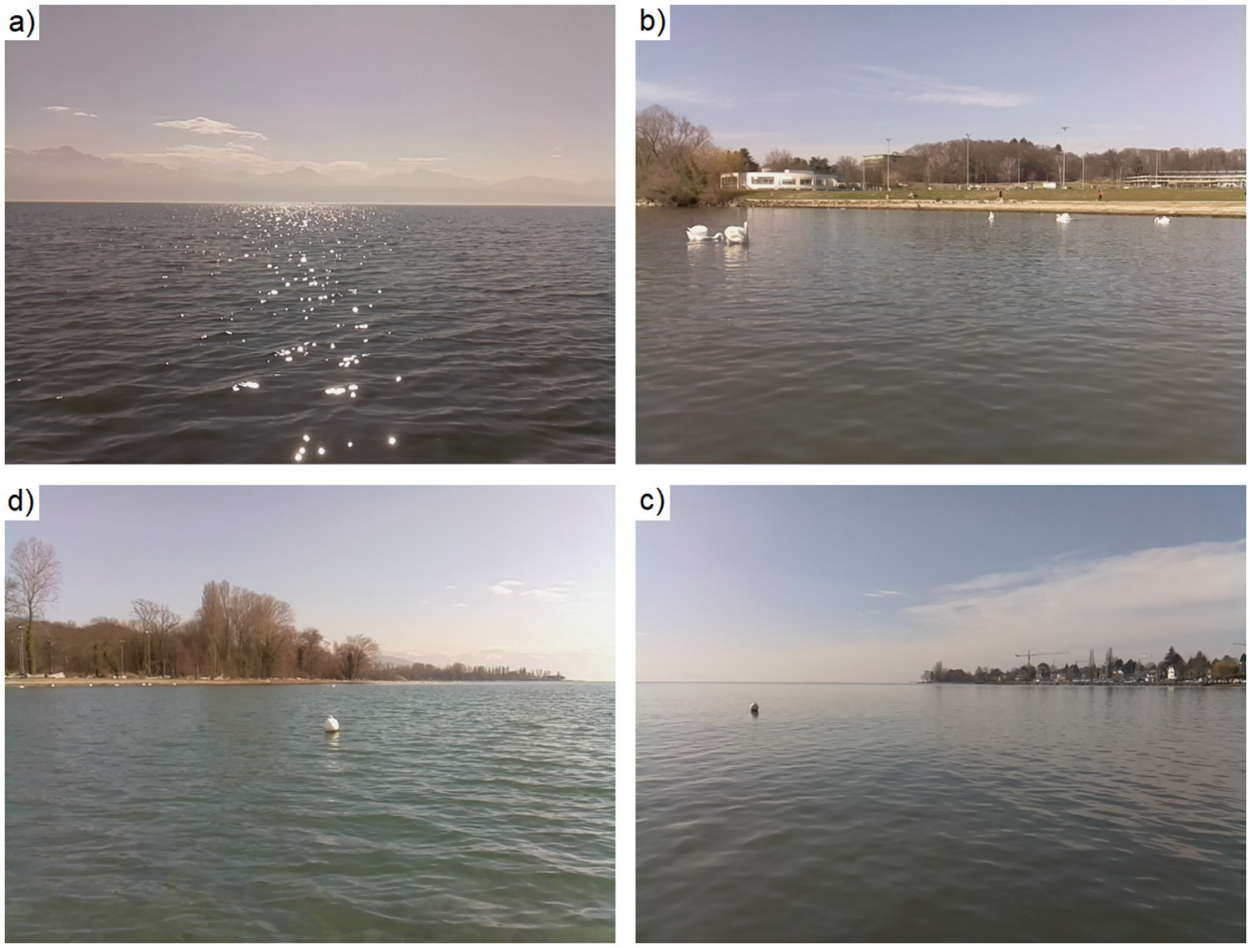
Typical images from different datasets used for horizon detection assessment. (a) Clear horizon with surface sun glint. (b) Close to shore with a small perspective effect. (c-d) Standard lake scenes with mixed close-, mid-, and long-range horizon.

We tested the efficacy of the horizon detection method by comparing it with a full-frame RANSAC detection, as proposed by Wang et al. [5]. Four image datasets were used, as shown in Fig 7. These datasets are examples of the different types of scene and conditions that could be encountered by an ASV under a range of lighting conditions during daylight hours. They total 1223 frames or about 5:31 min of recording (imaging at about 3.7 Hz).

**Fig 8:**
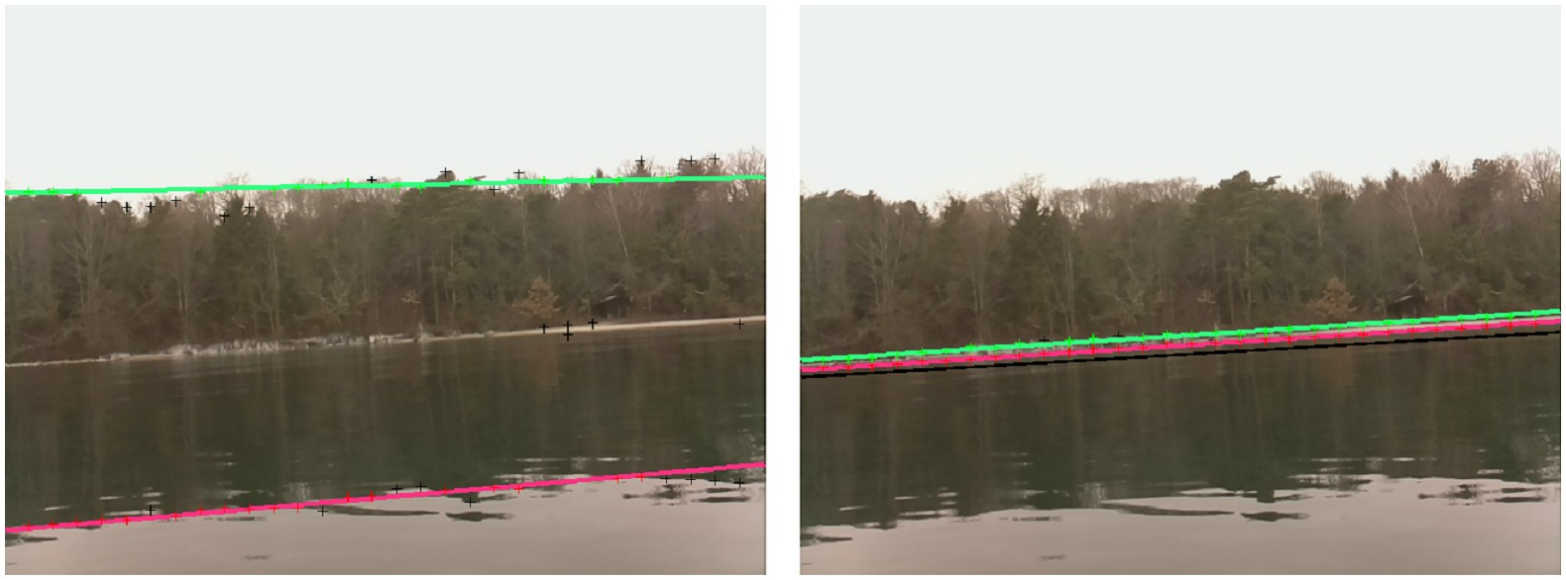
Accurate and inaccurate horizon detection comparison. The crosses correspond to the maximum gradient in the vertical stripes (not shown) for the RANSAC-only horizon detection considering the full-frame (a) and the restricted search window determined from the INS data (b). In both (a) and (b), the red line represents the detected horizon and the corresponding red crosses are the points correctly fitted by the line. (a) The detected water-land horizon is offset from its true position, and so is considered as inaccurate. (b) Improved horizon detection using INS data. The black line corresponds to the estimated horizon using the attitude of the ASV.

Note that, due to the proximity of the shore, not all the horizons shown in Fig 8 are straight. To be considered accurate, the detected horizon must not be offset by more than a few pixels and must be parallel to (at least a part of) the actual horizon. Fig 8 shows an example of both accurate and inaccurate detections taken from the most challenging dataset, Fig 8b.

Attitude-based (i.e., using INS for cropping then RANSAC) horizon detection achieves an accuracy of 99.51%, whereas full-frame RANSAC detection, such as proposed in [5], scores 71.83%. For the four datasets, Fig 8a–8d, individual (INS + RANSAC versus full-frame RANSAC) performances are 99.05%/100%, 100%/31.7%, 99.28%/88.07%, and 99.68%/67.82%, respectively. These results show that for the full-frame algorithm, performance is highly scene-dependent. It behaves poorly for nearby shore and corresponding high gradient frames, as seen in Fig 8, where attitude-based detection is less prone to undesired gradient detection during the vertical stripes analysis step. Also, for both methods, the fact that the horizon is assumed to be straight sometimes leads to poor results, but is still acceptable if a sufficient portion of the horizon is correctly detected. We recall that horizon detection is not important per se, rather that it is used to limit the subsequent analysis to the water surface. This goal is achieved with the presented method.

### Image segmentation

**Fig 9:**
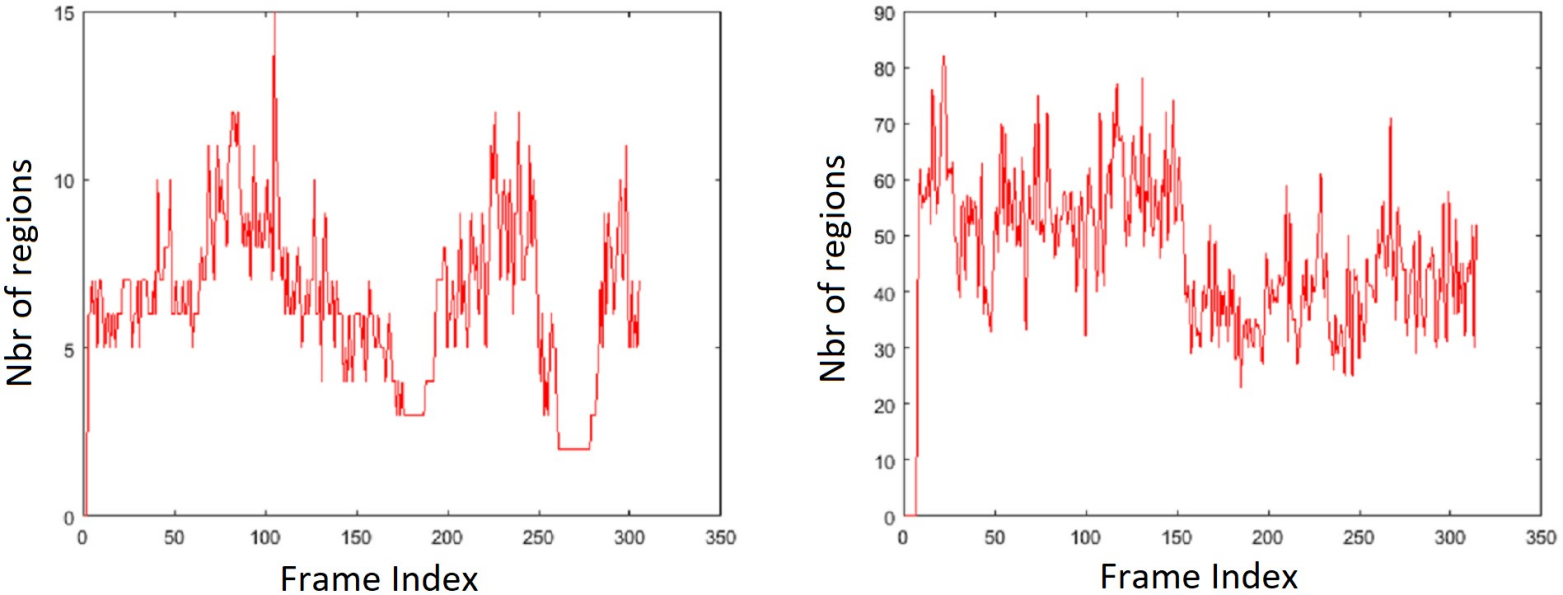
Glint detection results. Number of distinct regions after thresholding for DS4 (left) consisting of white obstacles and for DS7 (right) consisting of glint. Recall that the algorithm uses at least 20 distinct regions to identify glint-affected frames.

**Fig 10:**
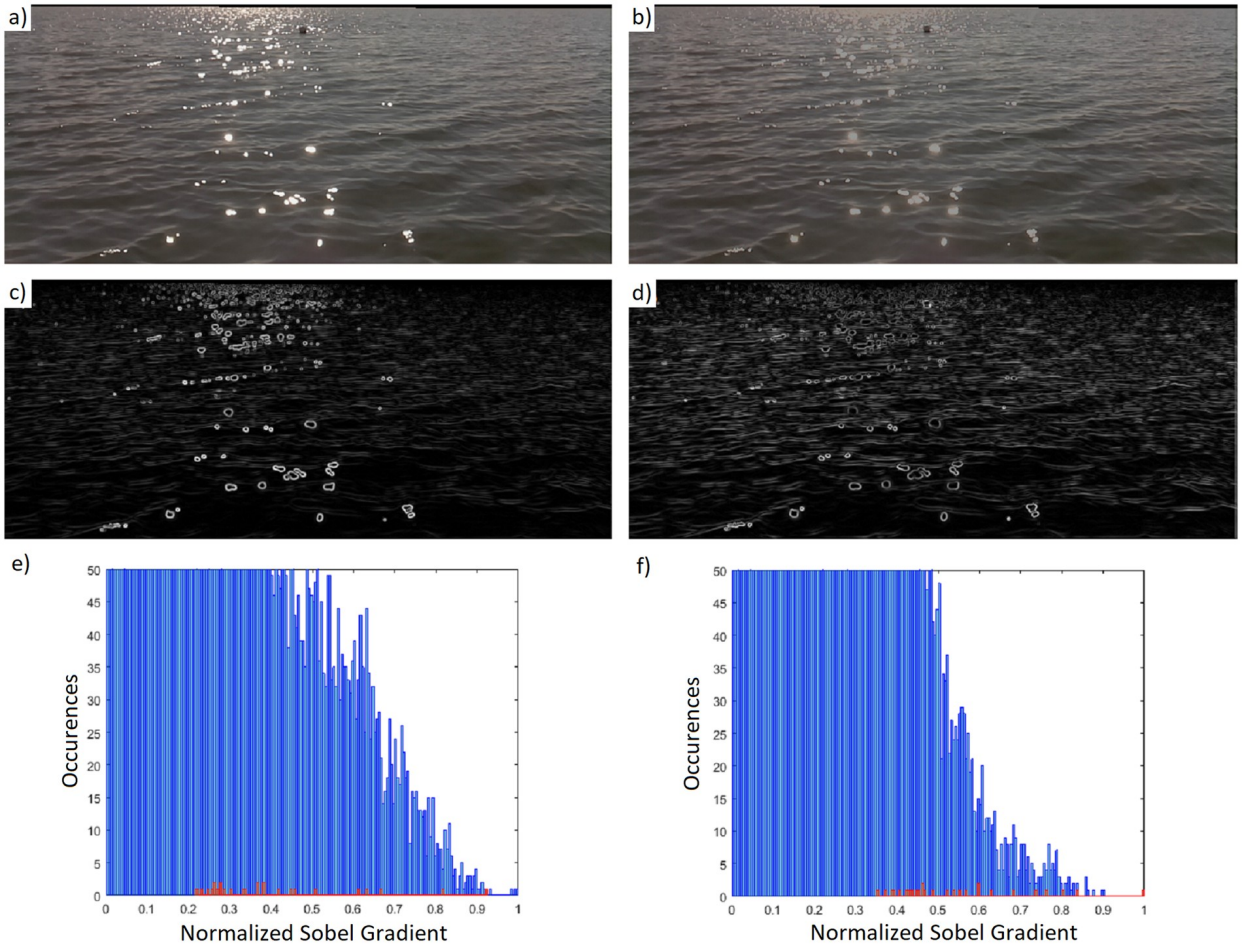
Glint reduction example. (a) Initial frame. (b) HSV value threshold applied. Threshold = 0.6. (c) Result of Sobel gradient operator on the initial frame. (d) Result of Sobel gradient operator after HSV value thresholding. (e) Histogram of Sobel operator gradient. Blue columns correspond to the whole cropped image, red columns to the buoy. (f) Histogram of Sobel operator gradient after algorithm application.

Before analyzing the image segmentation, we first consider the glint rejection step. As already mentioned, this step is applied only if glint is present in the frame. Fig 9 shows that glint can be distinguished from white obstacles accurately. We then show that the proposed glint reduction method efficiently reduces the gradient resulting from sun glint on the water surface. This allows for obstacle detection even under rough conditions when the obstacle is obscured by glint. In Fig 10a, a typical glint-affected frame is shown. In this case, the obstacle is a buoy with glint in both the foreground and background (note that the image is cropped to remove the previously detected water-land horizon). If we simply apply a Sobel gradient operation (Fig 10c), the buoy will not pass the following threshold operation because of the high gradient of the glint. This is also seen in the corresponding histogram (Fig 10e), where the buoy edge gradient is emphasized in red.

Now, if we first apply the glint reduction algorithm instead (Fig 10b) and then the Sobel operator, the result leads to detection of the buoy (Fig 10d). The glint-reduction step reduces the (original) high gradient of the glint, which causes a relative shift of the buoy edge gradient pixels inside the histogram, as seen in Fig 10f. The histograms show that, without glint reduction, the average buoy edge gradient magnitude is higher than 98.42% of the pixels of the frame. This value jumps to 99.32% with glint reduction. This is mostly due to a previously mentioned relative average gradient magnitude shift, from 0.394 to 0.506, considering a normalized gradient.

**Fig 11:**
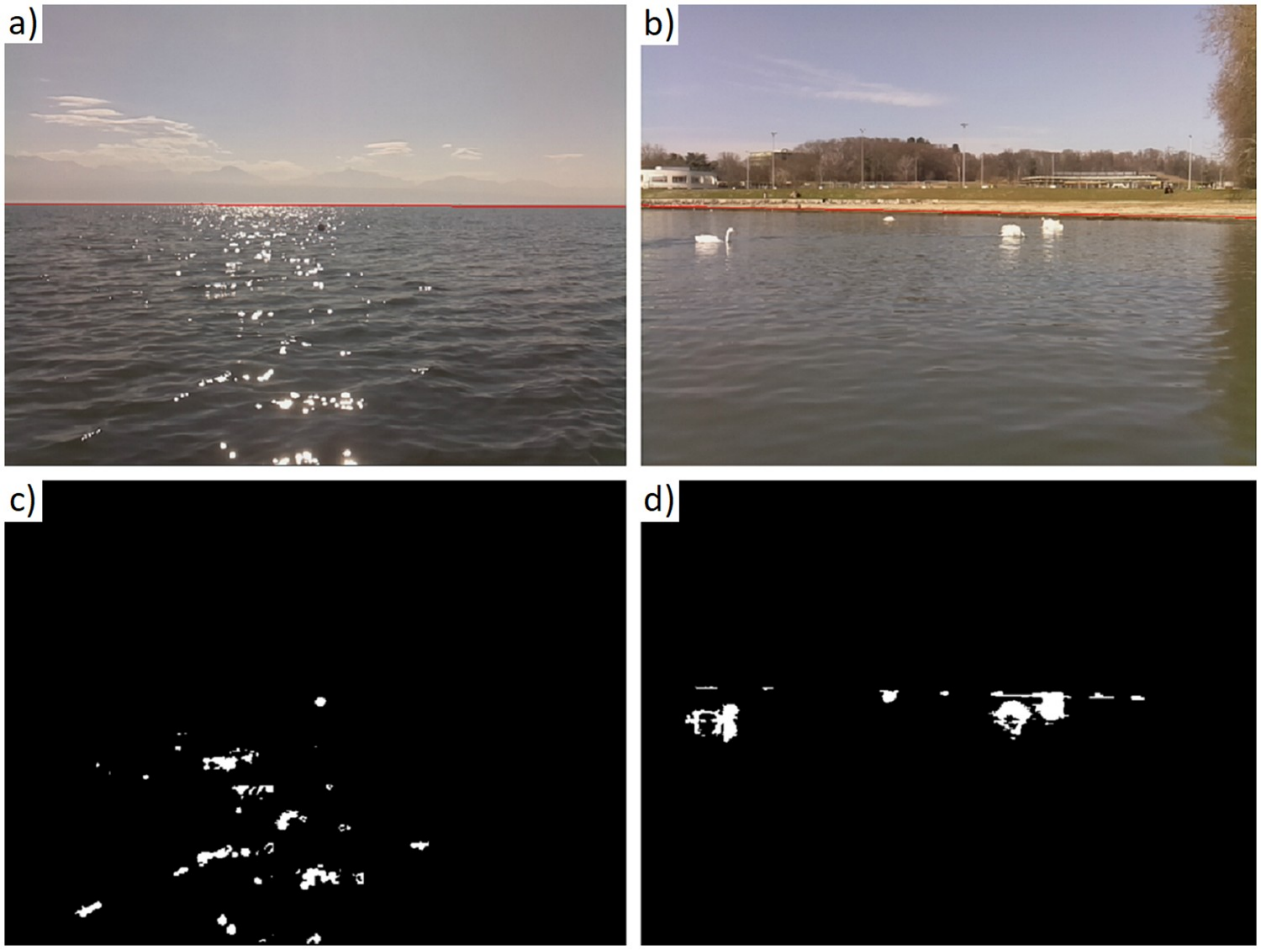
Segmentation results for two datasets. (a) Example frame from dataset DS7. (b) Example frame from dataset DS8. (c) Image segmentation for frame (a). (d) Image segmentation for frame (b).

The whole segmentation process performance was analyzed considering three challenging datasets, of which two example frames are shown in Fig 11, and two more standard datasets. The first of the challenging datasets, DS7 (Fig 11a & 11c), displays a buoy in front of sun glint. The glint reduction algorithm reduces the significance of glint and thus we can detect and segment the buoy accurately. The second and third datasets, DS4 and DS8 (Fig 11b & 11d), possess multiple obstacles that must be correctly individually segmented.

The three datasets comprise 556 frames containing at least one obstacle (buoy or swan). For the first dataset (307 frames), the buoy was correctly segmented in 242 frames (95.65%). In 9 frames, the buoy was only partially and not sufficiently segmented to be considered correctly detected. For the second and third datasets, the obstacles were swans. They were present in 303 frames, with an average of 3.78 swans per frame, of which 80.09% were correctly segmented. 12.14% were only partially segmented and thus considered wrong. The rest were missed. These three datasets gave the worst performance of the numerous cases examined. Table 3, a summary of the just-discussed datasets, also includes results for the two other datasets, totaling 263 frames with a single obstacle per frame in each. For these, the algorithm achieved 100% correct segmentation.

**Table 3:** Obstacle segmentation results. Values in parentheses are the ratio of partially segmented obstacles that can be considered either segmented or missed, as a proportion of the total number of obstacles.

Dataset	Segmented obstacles	Missed obstacles	Total nbr of obstacles
DS7	95.65% (10.28%)	4.35% (3.56%)	253
DS4 & DS8	80.09% (11.35%)	19.91% (12.14%)	1145
DS1	100% (0%)	0% (0%)	214
DS3	100% (46.94%)	0% (0%)	49

The aforementioned performance is the recall score (Eq 1). The precision score (Eq 2) is irrelevant since the algorithm forces 1.5% of the pixels to be segmented, even when no obstacles present. This increases the number of false positives, which are removed in the following step. The main goal here was not to miss obstacles.

### Multi-frame analysis

Segmented obstacles were compared across sequences of images (cropped above the horizon), and categorized as described above.

A margin of 2 pixels on each edge of each image was discarded. The side margins can contain image compression aberrations. The top margin can contain non-water elements due to small errors in horizon estimation. This could result in omission of obstacles lying on or just above the horizon. However, such objects are far from the ASV and hence not relevant. In addition, we ignored obstacles that covered less than 40 contiguous pixels. For a square object, this corresponds to a horizontal angle of 0.54°or an obstacle width size of about 4.7 cm at a distance of 5 m. Objects are considered actual obstacles only if they persist in at least 7 consecutive frames (about 2 s in our hardware setup), allowing for initial detection. Therefore, undetected obstacles that are seen for less than 7 frames will not be considered as false negatives.

For the 14 datasets considered for the analysis, we obtained a precision (Eq 2) of 94.66% and a recall (Eq 1) of 97.45% based on 2981 obstacles to detect, during a period of about 19 min (3342 frames). It took an average of 12 frames to detect an obstacle. Table 4 shows algorithm performance for each individual dataset. Fig 6 shows an example frame from the corresponding datasets.

**Table 4:** First part of full algorithm analysis results. Performance analysis for individual datasets.

Dataset	Frames	Obstacles	True Positive	False Negative	False Positive	Precision	Recall
DS1	327	85	85	0	0	100%	100%
DS2	313	0	0	0	0	–	–
DS3	230	158	147	11	0	100%	93.04%
DS4	158	152	152	0	0	100%	80.98%
		152	143	9			
		152	151	1			
		152	129	23			
		89	51	38			
		76	0	76			
DS5	122	82	79	3	0	100%	90.91%
		115	98	17			
		60	56	4			
		7	7	0			
DS6	238	137	135	2	0	100%	98.54%
DS7	307	161	155	6	0	100%	100%
DS8	144	138	76	62	26	91.39%	72.44%
		93	54	39			
		82	58	24			
		124	105	19			
		62	39	23			
		20	20	0			
DS9	201	0	0	0	112	0%	–
DS10	370	214	214	0	0	100%	96.89%
		75	66	9			
DS11	59	15	10	5	0	100%	63.01%
		43	21	22			
		15	15	0			
DS12	259	43	43	0	0	100%	100%
DS13	188	182	0	0	0	100%	100%
DS14	388	291	291	0	121	70.63%	100%
Total	3342	2981	2905	76	164	94.66%	97.45%

**Fig 12:**
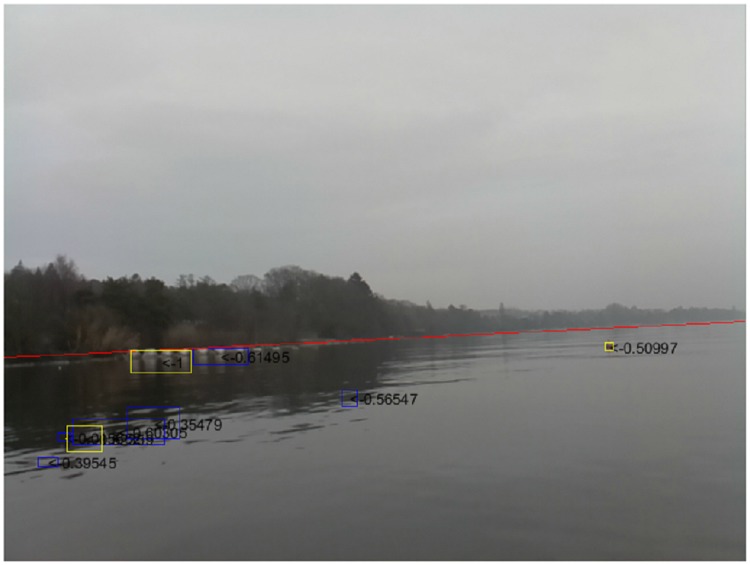
Example detection result in a challenging, low-light scene. Yellow rectangles represent detected obstacles (OBs), blue rectangles represent possible obstacles (POBs). The number next to each rectangle is the likelihood of either a POB or an OB. The red line corresponds to the detected horizon. In this case, we considered one false positive (bottom shore reflection), and one true positive (right buoy) only.

In the presented results, shore detection and its direct reflection are not considered as false positives, since the ASV should also avoid the shore (for instance, if the autonomous navigation developed a fault and the ASV headed towards the shore). On the other hand, an indirect shore reflection (i.e., blob not connected to the shore, or not detected until close to the ASV) is still considered to be a false positive, because in this case no direct obstacle threatens the ASV. Obviously, any other detection on the water not related to any physical object is considered a false positive. Fig 12 shows a detected obstacle and two detected shore reflections, one of which is considered as false positive. The top-left shore reflection is not considered a true positive as it is not an obstacle as defined above, and it is not considered a false positive as it still represents an effective obstacle.

## Discussion

We discuss the different steps of the algorithm separately.

- **Horizon detection**. The attitude-based RANSAC horizon detection gave better results than the standard full-frame RANSAC method, but at the cost of requiring an accurate attitude estimation. In particular, we noted that the INS data can be corrupted if the ASV is subjected to engine vibrations. In such a case, a higher quality INS unit would be necessary as the equipped BNO055 device, implementing a sensor fusion algortihm, does not allow to set a bandwidth filter for its integrated inertial sensors. An alternative would be mechanical damping of vibrations. Also, for the cropping around the estimated horizon we use the measured attitude error, which depends on the hardware used.
- **Glint reduction**. Glint is common in lake scenes, and detection of obstacles within glint is essential for safe ASV navigation. Our algorithm gives excellent results for this circumstance.
- **Image segmentation**. Image segmentation was proven to be effective. Our algorithm relies on gradient thresholding, meaning that low gradient obstacles could be missed. If a different camera is used, then we advise some tuning of the window size of the Gaussian filter used to compute the weight map.

The presented algorithm detects obstacles with a high degree of reliability. It performed very well, but a visual-based, single camera solution will never be 100% reliable. If desired, for a higher cost, performance improvements could be made with additional hardware components such as a LIDAR, second RGB camera for stereoscopy, or an infrared camera. With such additions, false positives could be discriminated and so the algorithm could be re-tuned to increase the recall score, at the cost of lower precision. The results obtained here were computed with parameters balancing both scores.

The presented algorithm involved some parameter tuning based on numerous test cases. These parameter values will possibly change for different hardware choices. The influence of the tunable parameters are summarized as:

- For the horizon detection, Eqs 3 and 4 can directly be computed for a particular setup geometry leaving only the maximum attitude error to be estimated for the interfaced INS system.
- The glint-reduction method involves three parameters, the initial fixed threshold, the minimum number of distinct segmented regions for the filter to be applied, and the maximum saturation value possible for individual pixels. The two initial parameters can be easily tuned by using an image with glint acquired with the desired setup. The threshold will mostly depend on the camera, wheras the minimum number of regions may slightly increase with the camera resolution. The threshold from Eq 5 should be set low enough to identify obstacles even when glint is seen.
- There are five parameters to be tuned for the image segmentation algorithm. The two first correspond to the percentile value of the initial threshold and the corresponding minimum allowed value for when no obstacles are seen in the frame. The percentile value should be tuned linearly with the resolution ratio. For instance, if the used resolution is 1280×960, instead of the presented 640×480, a good initial guess is to multiply the parameter by 2. The minimum threshold value must instead be tuned using frames without obstacles in different weather conditions. This allows estimation of the gradient magnitude resulting from wave patterns, and thus tuning of the threshold. The next parameter is the standard deviation of the Gaussian filter used to compute the weight map. This parameter should also scale up linearly with the resolution ratio. The last two parameters are the second threshold percentile and minimum value that can be tuned following the same methodology as for the first threshold. Finally, if necessary, the median filters could also be scaled with the resolution of the frames.
- Concerning the multi-frame analysis, parameters can be sorted into two categories. The first one encompasses parameters used for the matching of blobs. The only parameter of this category requiring tuning would be *dist*_*limit*_ from Eq 8 defining the radius around which a blob should remain in the following frame. Thus, this parameter not only depends linearly on the resolution of the optical system, but also has a linear dependence on the period between two consecutive frames. The higher the frame rate, the smaller the value of *dist*_*limit*_. The second category concerns the control of likelihood of tracked blobs. For higher frame rates, water patterns could more easily be detected as obstacles, thus the parameters controlling the update of likelihood of possible obstacle blobs (POBs) and obstacle blobs (OBs) must be tuned. This is achieved through reducing the value of *η* in Eq 10, since then more frames should be needed to transform a POB to an OB. With a higher frame rate, we can also assume that blobs will be more similar in two consecutive frames. We thus advise to increase the value of the thresholds and cost parameters, referred to as *confidenceThreshold* and *likelihoodCost* in Algorithm 3.

The algorithm was developed and tested on a large lake. An interesting extension would be to test its applicability on higher energy water bodies such as seas or oceans. However, we expect a priori that its performance may be degraded as the assumptions on which the segmentation algorithm is based are likely more applicable to relatively calm water.

## Conclusion

We presented a novel, efficient, and low-cost visual-based obstacle detection system for use on ASVs deployed on lakes. As presented, the device is standalone, is built from commercial off-the-shelf components, and can be deployed on different ASVs with minimal integration. Alternatively, full integration is possible using the ASV’s on-board computer and INS. Our solution performs the image processing and multi-frame analysis in-line at a (frame) rate of about 4 Hz, with obstacles detected typically within 2-3 s. It is well suited to slow-paced vehicles, a category that includes small-size ASVs. Because the time for obstacle detection is known, it is straightforward to estimate the algorithm’s appropriateness for faster vehicles. However, since the algorithm is tuned for the selected camera and its positioning on a particular ASV, we expect that some tuning would be beneficial if different hardware and mounting locations are used, recalling that best performance is expected for a (forward-pointing) camera mounted near the water surface. In the tested configuration, the algorithm achieved precision and recall scores, respectively, of 94.66% and 97.45%, obtained for 14 field-measured datasets totaling about 19 min of imagery (3342 frames).
